# Psychometric Properties of the Theory of Mind Assessment Scale in a Sample of Adolescents and Adults

**DOI:** 10.3389/fpsyg.2016.00566

**Published:** 2016-05-09

**Authors:** Francesca M. Bosco, Ilaria Gabbatore, Maurizio Tirassa, Silvia Testa

**Affiliations:** ^1^Department of Psychology, University of TurinTurin, Italy; ^2^Neuroscience Institute of Turin, University of TurinTurin, Italy; ^3^Faculty of Humanities, Research Unit of Logopedics, Child Language Research Center, University of OuluOulu, Finland

**Keywords:** Theory of Mind, Th.o.m.a.s., validation of ToM tests, social cognition, metacognition

## Abstract

This research aimed at the evaluation of the psychometric properties of the Theory of Mind Assessment Scale (Th.o.m.a.s.). Th.o.m.a.s. is a semi-structured interview meant to evaluate a person's Theory of Mind (ToM). It is composed of several questions organized in four scales, each focusing on one of the areas of knowledge in which such faculty may manifest itself: Scale A (I-Me) investigates first-order first-person ToM; Scale B (Other-Self) investigates third-person ToM from an allocentric perspective; Scale C (I-Other) again investigates third-person ToM, but from an egocentric perspective; and Scale D (Other-Me) investigates second-order ToM. The psychometric proprieties of Th.o.m.a.s. were evaluated in a sample of 156 healthy persons: 80 preadolescent and adolescent (aged 11–17 years, 42 females) and 76 adults (aged from 20 to 67 years, 35 females). Th.o.m.a.s. scores show good inter-rater agreement and internal consistency; the scores increase with age. Evidence of criterion validity was found as Scale B scores were correlated with those of an independent instrument for the evaluation of ToM, the Strange Stories task. Confirmatory factor analysis (CFA) showed good fit of the four-factors theoretical model to the data, although the four factors were highly correlated. For each of the four scales, Rasch analyses showed that, with few exceptions, items fitted the Partial credit model and their functioning was invariant for gender and age. The results of this study, along with those of previous researches with clinical samples, show that Th.o.m.a.s. is a promising instrument to assess ToM in different populations.

## Introduction

The aim of this study was to investigate the psychometric properties of the Theory of Mind Assessment Scale (Th.o.m.a.s.; Bosco et al., 2009), a semi-structured interview developed for the assessment of Theory of Mind (ToM) in adolescents and adults (healthy and with clinical pathologies). ToM is the capacity to ascribe mental states like emotions, intentions, desires, and beliefs to oneself and the others and to use this knowledge to predict, interpret, and explain the relevant actions and behaviors (Premack and Woodruff, 1978).

The classic tests for the assessment of ToM, the *false beliefs tasks*, were created in the domain of developmental psychology (Wimmer and Perner, 1983; Baron-Cohen et al., 1985). They require the subject to recognize another person's beliefs when they differ from those of the subject herself, under the assumption that this is the only certain proof of the availability of a theory of mind (Dennett, 1978). False belief tasks investigate first- or second-order ToM. The former (Wimmer and Perner, 1983) is the ability to understand a person's beliefs about a state of the world, whereas the latter is the ability to ascribe nested mental states, i.e., to understand a person's beliefs about someone else's beliefs (Perner and Wimmer, 1985). Empirical data have shown that children and clinical populations find second-order ToM tasks more difficult to solve than first-order ones (Mazza et al., 2001; Wellman and Liu, 2004). Due to the poor test-retest reliability for the scores obtained at false-belief questions, initial attempts to validate false belief tasks did not give fully satisfactory results (Mayes et al., 1996).

Only few studies have explored the psychometric properties of ToM tests. One is the Theory of Mind (TOM) test (Muris et al., 1999). This test, which was devised for children of 5–12 years, is an interview composed of vignettes, stories, and drawings about which the child is asked to answer several questions. The test was administered to a sample of children with developmental disorders and a healthy one, showing that it is able to discriminate between the two conditions and that its scores have good internal consistency and inter-rater reliability, and sufficient test-retest stability.

Other ToM tasks, like the Strange Stories (Happé, 1994) and the Faux pas (Baron-Cohen et al., 1999), were created to evaluate more sophisticated aspects of ToM in children older than four years of age. The Strange Stories task (Happé, 1994) assesses the comprehension of complex mental states like misunderstanding and double bluffing, which require understanding social contexts. It has been used with children, both in healthy (e.g., Devine and Hughes, 2013) and pathological conditions (e.g., Charman et al., 2001; Kaland et al., 2002; Velloso et al., 2013) as also in adolescents and adults with Autism Spectrum Disorders (ASD) and Asperger syndrome (Jolliffe and Baron-Cohen, 1999; Kaland et al., 2005),

Although the tests discussed so far were first created for use in developmental psychology they have often been employed, possibly with some adaptation, in adults with clinical disorders like schizophrenia (see for example Mazza et al., 2001; Pickup and Frith, 2001) in addition to other specific tests, mostly involving picture sequencing tasks (see for example Langdon et al., 2001; Brüne and Bodenstein, 2005; Brüne et al., 2016). To our knowledge, however, only few psychometrics evaluations of these tests in healthy adults have been provided. One is the widely used Reading the Mind in the Eyes task (RME; Baron-Cohen et al., 2001) originally created to assess ToM in children with Asperger Syndrome. It consists of photographs of the eyes region: the subject is asked to match each picture with the semantic definition of a specific emotion (e.g., “worried,” “annoyed”). Several studies of the psychometric properties of the RME were conducted with healthy adults in different countries, but not with unanimous results: some studies found a low level of internal coherence (Voracek and Dressler, 2006; Harkness et al., 2010; Olderbak et al., 2015) whereas others found an acceptable one (Serafin and Surian, 2004; Vellante et al., 2013). Reports of test-retest reliability RME scores range from acceptable (Yildirim et al., 2011) to good (Vellante et al., 2013). RME is commonly used to assess ToM; however, because judgments are only based on eyes expression, it only focuses on a specific kind of mental state, namely recognition of emotions, and therefore is able to assess only one facet of ToM.

Recently, more attention has been paid to psychometric properties in the creation of novel ToM tests. These tests have mainly been designed to investigate ToM in children with ASD. For example, the Animated Theory of Mind Inventory for Children (ATOMIC; Beaumont and Sofronoff, 2008) was created to assess ToM in children with Asperger Syndrome. The tool consists of cartoons depicting a range of themes, each followed by two multiple-choice questions. The ATOMIC has proved capable of discriminating between clinical and control groups and appears to be significantly correlated with the Strange Stories task (Happé, 1994). Also the Theory of Mind Inventory (Hutchins et al., 2012) was developed to assess ToM in individuals with ASD. It works by asking the parents to compile a questionnaire consisting of statements toward which the interviewee expresses agreement or disagreement on a continuous metrics. The instrument appears to have excellent test-retest reliability and internal consistency. Another recently developed tool, created for children with high functioning ASD is the Comic Strip Task (CST; Sivaratnam et al., 2012). It consists of vignettes investigating the child's comprehension of other persons' beliefs, intentions, and emotional states and it appears to have moderate internal consistency and good discriminant validity.

A different set of clinical tools, specifically created for adults, investigates different, albeit related cognitive ability, namely self-reflection (Fonagy et al., 1991), and metacognition (Semerari et al., 2003). Self-reflection is the capacity to understand and reason upon one's own and other's states like feelings, thoughts, fantasies, beliefs, and desires (Gergely et al., 2002). Fonagy et al. (1998) developed the Reflective Functional scale (RF) to study the subjects' ability to reflect upon their childhood experience in mentalizing terms. The coding for the RF is based on the interviewee's ability to reflect on several relevant passages of the Adult Attachment Interview (Main and Goldwyn, 1990). Despite the possible theoretical similarity between the notion of self-reflection, as investigated by the RF scale, and that (or those) of ToM, a study of Taylor et al. (2008) conducted with persons with autism, failed to find significant correlations between their performance on the RF and ToM, at least as assessed with the RME test discussed above (Baron-Cohen et al., 2001). Most studies available in the literature that use the RF are based on the Adult Attachment Interview; however, recent researches have applied the RF to other clinical interviews, e.g., the Brief Reflective Functioning Interview (BRFI; Rudden et al., 2005) and the Reflective Functioning Rating Scale (RFRS; Meehan et al., 2009). Moreover, in a recent review on Reflective Functioning Katznelson (2014, p. 115) concluded that “more research regarding reliability and validity of these measures -BRFI and RFRS- is necessary to qualify these more thoroughly.” Still another limitation of the RF is that it yields a unique total score, thus underestimating the complexity of mentalizing activities (Choi-Kain and Gunderson, 2008; Gullestad and Wilberg, 2011).

Metacognition is a wider construct. In Flavell's (1979) original definition, it includes any thought process that has as its object the mind itself in its various interpersonal, emotional, and cognitive dimensions. Examples of metacognition are memory, perception, or motivation. To study it, Semerari et al. (2012) developed the Metacognition Assessment Interview (MAI), a semi-structured interview aimed to investigate different aspects of metacognition; MAI is an adaptation of the Metacognition assessment Scale (MAS; Semerari et al., 2003). Semerari et al. (2012) investigated the psychometric proprieties of the MAI on a sample of non-clinical subjects. Factors analysis showed a two factors hierarchical structure corresponding to the two main metacognitive functions, the “self domain,” which is the ability to monitor and integrate mental aspects and the way in which a person is aware of her mental state in relation to her behavior, and the “other domain,” which is the ability to adopt another person's perspective and to differentiate between different forms of representations, such as imagination, expectations, and reality. The inter-rater reliability and the internal consistency of MAI in these two domains were acceptable (Semerari et al., 2012).

Despite being obviously related to ToM, metacognition is a wider construct, including more sophisticated mental functions (Semerari et al., 2003, 2012) than the former, originally considered by Premack and Woodruff (1978) as a unitary faculty. Accordingly, most available tools for assessing it have embedded this assumption into their methodological approach and material structure. In time, however, it has been argued that ToM has a much more complex nature, thus opening the way to the possibility of decomposing it into different aspects or components.

A first such operation is the distinction between *third-person ToM*, i.e., the ability to attribute mental states to another person, and *first-person ToM*, i.e., the ability to attribute mental states to oneself (Nichols and Stich, 2003; Dimaggio et al., 2008). To understand oneself and to understand another person appear to be different activities, mediated by different processes and recruiting different kinds of knowledge. Within the domain of third-person ToM a further distinction, proposed by Frith and De Vignemont (2005), takes place between an *egocentric* and an *allocentric* perspective. In the former, the mental states of other agents are represented in relation to the self, while in the latter they are represented independently from the self. Still another difference occurs between *first-order* and *second-order* ToM. First-order ToM is the ability to grasp someone's mental states (Wimmer and Perner, 1983), while second-order ToM is the ability to infer what someone thinks about a third person's mental states (Perner and Wimmer, 1985). Studies in the developmental (Wellman and Liu, 2004) and in the clinical domains (e.g., in patients with schizophrenia, Mazza et al., 2001) show that first-order tasks are easier to be solved that second-order ones.

Further differences may be drawn between different types of mental states that can be dealt with by the agent. It is commonly theorized in other areas of cognitive science that at least three such types, namely *beliefs, desires*, and *intentions*, are needed to capture an agent's mind (see e.g., Rao and Georgeff, 1992; Tirassa, 1999; Tirassa and Bosco, 2008), and theories in developmental psychology also point to the idea that the comprehension of volitional and epistemic states may be acquired at different ages (e.g., Wellman, 1991; Wellman and Liu, 2004). Furthermore, it might be sensible to distinguish between different ways to which ToM may be put to use, e.g., in understanding or predicting another agent's behavior, in attempting to affect it, and so on.

The Th.o.m.a.s. (Bosco et al., 2009) is a semi-structured open-question interview devised to capture these various facets of ToM, namely first vs. third person, first vs. second order, egocentric vs. allocentric, different kind of mental states and different uses that can be made of them, and thus to provide a broad assessment of ToM abilities both in healthy (adolescents and adults) and clinical conditions. Having a single instrument capable of assessing several different facets or components of ToM allows to directly compare how they function in the same individual or clinical sample.

Th.o.m.a.s. has been used in patients with a diagnosis of schizophrenia (Bosco et al., 2009), preadolescents and adolescents (Bosco et al., 2014b), sex offenders (Castellino et al., 2011), persons with alcohol use disorder (Bosco et al., 2014a), persons with congenital heart disease (Chiavarino et al., 2015), and persons with bulimia (Laghi et al., 2014). In all these types of subjects Th.o.m.a.s. has systematically proved a useful clinical tool, capable of discriminating between healthy control and non-healthy participants. Furthermore, it keeps into account that different kinds of patients may in principle, and actually do in practice, show different patterns of performance to the various ToM components mentioned above. In particular, persons with a diagnosis of schizophrenia, persons with alcohol use disorder, and sex offenders (Bosco et al., 2009, 2014a; Laghi et al., 2014), in comparison to healthy controls, were impaired to all the ToM dimensions investigated. Persons with bulimia showed impairment in third-person ToM in the allocentric perspective and in second-order ToM, but not in third-person ToM in the egocentric perspective or in first-order ToM. Finally, persons with congenital heart diseases showed impairment to third-person ToM, both in the egocentric and the allocentric perspective, but not in first-person or second-order ToM (Chiavarino et al., 2015). Globally, these studies testify to the necessity to have a tool able to separately investigate different ToM dimensions in clinical samples.

With the aim of verifying whether the results from Th.o.m.a.s. could be explained merely by differences in communicative-pragmatic abilities, Bosco et al. (2014b) created a second set of criteria for the evaluation of the participants' performance. The findings showed that communicative-pragmatic abilities, at least for the level required to answer Th.o.m.a.s., do not affect performance.

The goal of this research is to further investigate the validity of Th.o.m.a.s. by assessing its reliability, its dimensional structure and some aspects of items functioning and criterion validity in a sample of healthy people. In particular, we expect to find a fair to good inter-raters reliability and a good internal consistency. We also expect to find a correlation between Th.o.m.a.s. Scale B and another ToM task, the Strange Stories (Happé, 1994; Mazzola and Camaioni, 2002), because both tasks investigate third-person ToM in an allocentric perspective. For what concerns the dimensional structure we expect to find four dimensions corresponding to the four scales, namely first-person ToM (Scale A), third-person allocentric ToM (scale B), third-person egocentric ToM (Scale C) and second-order ToM (Scale D), and an invariant functioning of items across gender and across age groups.

## Materials and methods

### Participants

Two nearly equal-sized samples of preadolescent/adolescent and adult volunteers, all native speakers of Italian, were recruited in a number of local schools, university faculties, social organizations, sports clubs in two Italian cities (Torino and Asti). All the participants took part voluntarily in the study; all of them, as well as their parents when underage, were informed about the procedures and gave their informed consent. The study was approved by the Bio-ethical Committee of the University of Turin.

None of them resulted to have a history of significant neurological and/or psychiatric disorders or drug or alcohol abuse. During the recruitment phase, an assistant to the research (with a degree in Psychology) handed to the prospective participants an informative letter explaining the goal of the research. The letter also asked the subjects to withdraw from the study if they did not feel like participating or in the event of a past history of neurological or psychiatric disease, current or past history of alcohol or drug abuse, and current or past history of a psychotherapy.

The preadolescents and adolescents sample was composed of 80 participants (42 females), ranging in age from 11 to 17 (*M* = 14.0; *SD* = 2.25), with an education ranging from 5 to 12 years (*M* = 8.53; *SD* = 2.3). The adults sample consisted of 76 individuals (35 females), ranging in age from 20 to 67 years (*M* = 40.72; *SD* = 11.93) with an education ranging from 5 to 18 (*M* = 12.16; *SD* = 4.27).

Two participants were excluded from the analysis due to technical problems with the audio recording of the interview.

## Materials

### Theory of mind assessment scale (Th.o.m.a.s.)

Th.o.m.a.s. (see the references above) consists of 37^1^ open-ended questions that ask the interviewee to present and discuss her reflections about the functioning of ToM in everyday life (see Appendix A in Supplementary Material for the complete list of items), also with the aid of examples that she may provide spontaneously or after a specific request from the interviewer.

The architecture underlying the interview groups the questions in four scales that focus on the various internal or social domains in which ToM plays a role.

- Scale A (I–Me)—First-order first-person ToM. It focuses on how the interviewee (I) reflects on her own mental states (Me).
- Scale B (Other–Self)—Allocentric third-person ToM. These questions focus on how the interviewee thinks that other persons (Other) reflect on their mental states (Self), independently on her own position. This scale is akin to classic third-person ToM task.
- Scale C (I–Other)—Egocentric third-person ToM. These questions focus on how the interviewee (I) reflects on the mental states of other actors (Other). While both scales B and C investigate third-person ToM, the difference is that here it is the interviewee's positions that are highlighted, thus providing a sort of bridge between first- and third-person ToM.
- Scale D (Other–Me)—Second-order first-person ToM. These questions focus on how the interviewee conceives of the knowledge that the others may have of her mental states, that is how they (Other) reflect on her mental states (Me). The abstract structure of these questions thus is akin to classic second-order tasks.

The four scales are each divided into three subscales investigating Awareness, Relation, and Realization, that is, respectively, how the interviewee perceives different types of mental states, how he recognizes the causal relations that hold between these mental states and between them and an agent's visible behaviors, and how he conceives of the possibility of affecting the mental states of his own and those of the others. The types of mental states investigated are the most basic that must be comprised in a complex cognitive architecture (Olson et al., 2006; Tirassa et al., 2006a,b; Tirassa and Bosco, 2008), namely positive and negative emotions, volitional states like desires and intentions, and epistemic states like knowledge and beliefs.

The replies given by the interviewee are organized into a grid (Table 1) of which the scales and subscales are the columns and the types of mental states investigated are the rows. Each cell is thus located at the intersection of two of the dimensions considered, and each question, focusing on a specific aspect of the features of ToM, refers to one cell of the table.

**Table 1:** **A graphic representation of the structure of Th.o.m.a.s**.

**Scale**	**A (I–Me) First-order first-person ToM**			**B (Other–Self) Allocentric third-person ToM**		
**Subscale**	**Awareness**	**Relation**	**Realization**	**Awareness**	**Relation**	**Realization**
Beliefs	x	5	10	x	15 (15a)	20
Desires	7 (7a)	8 (8a)	9	17 (17a)	18 (18a)	19
Positive emotions	1 (1a)	2	6 (6a)	11 (11a)	12	16 (16a)
Negative emotions	3 (3a)	4	x	13 (13a)	14	x
**Scale**	**C (I–Other) Egocentric third-person ToM**			**D (Other–Me) Second-order first-person ToM**		
Beliefs	x	25 (25a)	28	x	35 (35a)	38
Desires	29	26	x	39	x	x
Positive emotions	21 (21a)	22	27	31 (31a)	32	37
Negative emotions	23 (23a)	24	x	33 (33a)	34	x

*Numbers in the table (e.g., 1) refer to the same-numbered question; numbers in parentheses (e.g., 1a) refer to the “Why not version” of the same question; for example, if the subject responds negatively to question [1]: “Do you ever feel emotions that make you feel good?” the interviewer poses question [1a]: “Why not?”. Some cells contain an (x) because not all the intersections between two dimensions have a relevant question, since in some cases this would sound contrived. For example, no question asking whether the interviewee is aware of his own beliefs is posed, as it may be assumed that if one were not, one would just be unable to talk about them. Adapted from Bosco et al. (2009)*.

For example, question [3]: *When you feel bad, do you understand the reason why you feel like that?* explores how the interviewee reflects on her own negative feelings (dimensions investigated: Awareness and Negative emotions); question [18]: *Do the others try to fulfill their desires?* asks the interviewee to reason about how the others' desires and feelings are interconnected (dimensions investigated: Relation and Desires); and so on for each question.

### Strange stories

In addition to Th.o.m.a.s., the participants were also administered a selection of six items from the Italian version of the Strange Stories (Mazzola and Camaioni, 2002), originally devised by Happé (1994). Each story contains two test questions: the comprehension question (e.g., *Was what X said true?*), and the justification question(s) (e.g., *Why did X say that?)*. The latter question requires an inference about the speaker's/actor's intentions; correct performance requires attribution of mental states such as desires, beliefs or intentions, and sometimes higher-order mental states such as one character's belief about what another character knows.

### Procedure

The participants completed the Th.o.m.a.s. interview and Strange stories task individually with a research assistant. The material was administered at school (adolescents) or at home (adults); the session generally takes about 1 h. The research assistants participating in the research were in total three. They all had a degree in psychology and were trained by two of the authors (I.G. and F.M.B.) on how to administer the interviews. First they received an oral explanation of the aim and the procedure for the administration of Th.o.m.a.s. by I.G. or F.M.B. They then practiced in the administration of Th.o.m.a.s. to a test subject (not included in the experimental sample) and transcribed the interview. The transcription was then examined by I.G. or F.M.B.: if it was not satisfactory (e.g., because the interviewer had suggested one or more answers), the error was demonstrated and explained and another test interview was conducted (again the subject was not included in the sample). The procedure was repeated until the interview was conducted satisfactorily (two/three test interviews always did the job).

With the authorization of the interviewees or of their parents all the interviews were tape-recorded and then transcribed to enable offline scoring. The participants were informed that their participation was voluntary and that the aim and contents of the research would be explained at the end of the session.

The responses both to Th.o.m.a.s. and to the Strange Stories were rated by another research assistant, blind to the aims of the study; moreover, 29% of the sessions were rated by a second independent judge, again blind to the aim and the scope of the research, in order to evaluate the inter-rater agreement. In rating Th.o.m.a.s. the judges were instructed to assign each answer a score from 0 to 4, according to given rating criteria (see Appendix B in Supplementary Material), and to insert it in the relevant cell of the scoring grid.

In rating the Strange Stories task the judges followed the criterion originally proposed by Happé (1994), namely to assign 0 to an incorrect answer and 1 to a correct one. A score of 1 was attributed when the individual replied correctly to both the comprehension and the justification question.

The inter-rater reliability among the scores assigned by the two independent judges at the Strange Stories task was calculated using Intraclass Correlation Coefficent; the ICC was 0.94, indicating a very high agreement between raters.

### Data analysis

The averages of the scores at each Th.o.m.a.s. scale and those of the Strange Stories task were inserted in the dataset and used for part of the analysis.

In order to assess the inter-rater agreement an Intraclass Correlation Coefficient^2^ (ICC) was calculated on the 29% of the sample for which the Th.o.m.a.s. interviews had been encoded by two judges. As a rule of thumb, values between 0.41 and 0.60 stand for fair reliability, those between 0.61 and 0.80 for moderate reliability, and those between 0.81 and 0.90 for substantial reliability (Shrout, 1998). Cronbach's alpha was used to evaluate the internal consistency of the scores on the four scales.

Confirmatory factor analysis (CFA) was applied to assess the goodness of fit of the 4-factors model representing the four scales, namely A (I–Me), B (Other–Self), C (I–Other), and D (Other–Me). The analysis was performed on the covariance matrix of the 37 items (Appendix C), using Lisrel 8.72 (Jöreskog and Sörbom, 1996). Because of the small size of the sample, Maximum Likelihood method (ML) without correcting the chi-square and standard errors was employed even though data violated the multinormality condition (Mardia's multivariate omnibus test of skewness and kurtosis (2, 154) = 1711.8; *p* < 0.001). The following criteria were used to evaluate the fit of the model as acceptable: RMSEA < 0.08; CFI > 0.95; SRMR < 0.08 (Browne and Cudeck, 1993; Hu and Bentler, 1995, 1999). In order to assess whether the tasks composing the four scales require different levels of ToM ability the Friedman test was performed on the four average scores.

A Rasch model for items with ordered response categories, the Partial credit model as implemented in Winsteps (Linacre, 2009), was applied to assess the psychometric properties of each unidimensional scale. Dimensionality was checked by performing Principal component analysis (PCA) on the residuals; scales for which the first eigenvalue was ≤ 2 were considered unidimensional (Linacre, 2009). Scores reliability was evaluated by the Person Separation index (PSEP), where values ≥1.50 are considered acceptable (Boone et al., 2014). Item quality was assessed by Infit and Outfit statistics and values within the 0.7–1.3 range were considered satisfactory (Wright and Linacre, 1994). Differential item functioning (DIF) for gender and age groups was evaluated: a DIF value > 0.64 logits (in absolute value) with a *p* < 0.05 was considered indicative of the persistence of a difference in item functioning across gender or age groups, after controlling for differences in person location (Boone et al., 2014).

Criterion validity was assessed as the difference in means between adolescents (whose scores were expected to be lower) and adults (whose scores were expected to be higher) and as the correlation with the independent evaluation of ToM provided by the Strange Stories. Multivariate analysis of variance (MANOVA) was employed to assess the difference in means between adolescents and adults on the four scales. Such approach was needed because of the high correlation between the dependent variables. Since the two groups were about the same size, both multivariate and univariate tests could be considered robust to departures from normality and from homogeneous covariance matrices conditions^3^. To assess the correlation between the scores at the Th.o.m.a.s. and that at the Strange Stories task the Pearson coefficient partialized for age and years of education and unpartialized was calculated.

With the exception of CFA and Rasch analysis, all the analysis were performed with SPSS 20.

## Results

### Inter-rater agreement and internal consistency

Overall, the inter-rater agreement was acceptable (Table 2). In particular, scale A (first-person ToM: I–Me) and scale D (second-order ToM: Other–Me) displayed fair reliability (0.59 and 0.49 respectively) whereas scales B and C, respectively investigating allocentric third-person ToM (Other–Self) and egocentric third-person ToM (I–Other), showed moderate reliability (0.65 and 0.71, respectively). All the four scales provided good results for internal consistency. Cronbach's alpha ranged from 0.86 to 0.89 (Table 2).

**Table 2:** **Inter-rater agreement (***N*** = 45) and internal consistency (154) of the four Th.o.m.a.s. scales**.

**Scale**	**ICC**	**alpha**
A (I–Me) First-order first-person ToM	0.59	0.89
B (Other–Self) Allocentric third-person ToM	0.65	0.88
C (I–Other) Egocentric third-person ToM	0.71	0.89
D (Other–Me) Second-order (first-person) ToM	0.49	0.86

### Factorial structure

**Figure 1:**
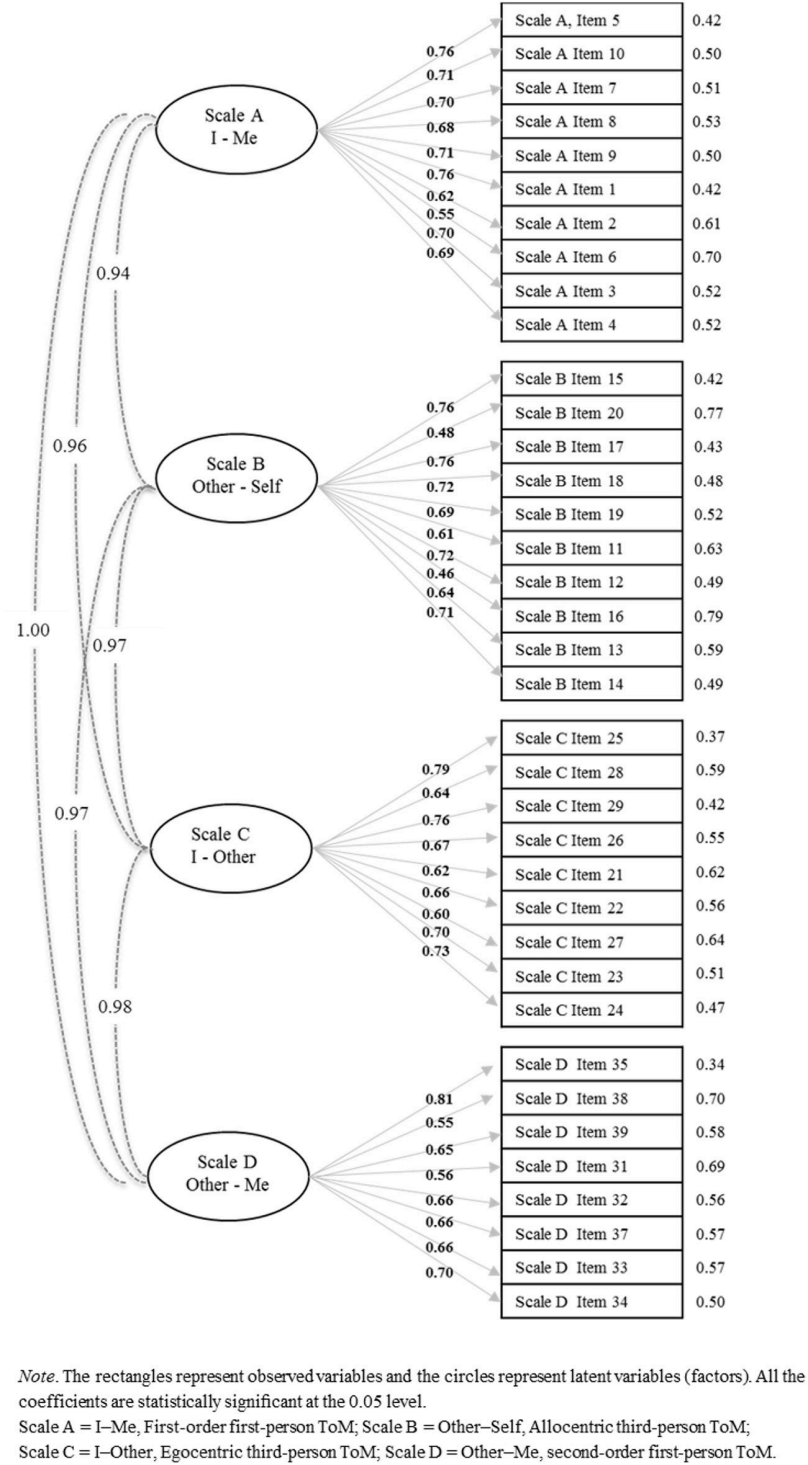
**Standardized solution of the four-factors CFA model of Th.o.m.a.s. (***N*** = 154)**.

The theoretical model consisting of four latent variables representing the four Th.o.m.a.s. scales fitted the data quite well: $\chi_{\left(623\right)}^{2}=1138$.4, *p* < 0.001; RMSEA = 0.073 (CI 90% = 0.066–0.080); CFI = 0.97 and SRMR = 0.058. All the loadings were high and statistically significant (Figure 1). The correlations between the four dimensions were very high, ranging from 0.94 to 1.00.

In order to assess which of the four scales can be discriminated in a healthy sample, several more parsimonious models with 3, 2, and 1 factors were estimated; the chi-square difference test and the Consistent Akaike Information Criterion (CAIC) were used to compare nested and non-nested models respectively. The bidimensional model that isolated the Scale B (Other–Self) resulted in a statistically nonsignificant χ^2^ difference test when compared to the 4-factors solution [$\chi_{\left(5\right)}^{2}=9.6$; *p* > 0.05] and lower CAIC value; consequently, it was chosen as the model which fitted the data best. In this solution with $\chi_{\left(628\right)}^{2}=1148$.0, *p* < 0.001; RMSEA = 0.073 (CI 90% = 0.066–0.080); CFI = 0.97, and SRMR = 0.058, all the loadings were statistically significant and high, ranging from 0.59 to 0.81 (with a mean of 0.66 and 0.67 for the two factors) and the standardized covariance was equal to 0.96. The unidimensional model, albeit adequate in terms of fit indices, was not acceptable because it exhibited a significant χ^2^ difference test when compared to the 4-factors solution [$\chi_{\left(6\right)}^{2}=20.8$; *p* < 0.01]. Thus, in a healthy sample, only two factors seem distinguishable: the one belonging to Scale B (Other–Self) and a broader one composed by the other three scales.

In order to investigate whether differences in the performance at the different scales were detectable, we analyzed the means of each scale. On average, the sample performed better on scale A (I-Me: *M* = 3.50; *SD* = 0.49) than B (Other-Self: *M* = 3.32; *SD* = 0.55), C (I-Other: *M* = 3.31; *SD* = 0.58) or D (Other-Me: *M* = 3.30; *SD* = 0.55): the Friedman test resulted in a significant overall effect [$\chi_{\left(3\right)}^{2}$ = 77.2; *p* < 0.001] and the post-hoc analysis, with a Bonferroni correction applied, showed that only the pairwise comparisons involving scale A (I-Me) were statistically significant (*p* < 0.001).

### Rasch analysis

Considering the high correlations between the four factors yielded by the CFA analysis, the Partial credit model was estimated on the whole pool of 37 items. The PCA on residuals signaled that more than one dimension were present as the eigenvalues of the first three components were >2. Excluding scale B, that resulted as a separate factor in the previous CFA analysis, the eigenvalue criteria was not yet respected since the first and the second eigenvalues were still >2. Therefore, the four scales were analyzed separately, which yielded the results summarized in Table 3.

**Table 3:** **Summary of the Partial credit model results**.

**Sub-scales**	**PCA (a)**	**PSEP (b)**	**Infit (c)**	**Outfit (c)**	**DIF (d) sex**	**DIF (d) age**
A	1.9	1.83	Item 2 Item 6	Item 2 Item 6 Item 1	Item 4	
B	2.1	1.89	Item 16 Item 20	Item 16 Item 20 Item 18	Item 20	Item 16 Item 17
C	1.7	1.99	–	–	–	Item 23 Item 26
D	2.1	1.82	–	–	–	Item 38

*(a) First eigenvalue of the Principal component analysis on residuals; (b) Person separation index; (c) items with infit/outfit statistics out of the range 0.7–1.3; (d) Items with differential functioning for sex or age (adolescents vs. adults)*.

Eigenvalue criteria were respected for scales A and C, slightly above the cut-off for scales B and D. Reliability was good for all the scales, giving scores with PSEP values > 1.5. All the items of scales C and D showed acceptable values for Infit and Oufit statistics and their functioning was invariant for gender. The two scales resulted to be partially invariant with respect to age groups: two items of scale C and one of scale D had a non-negligible DIF value between adolescents and adults. Some misfitting items were present in scales A and B; these scales exhibited partial invariance for both gender and age groups. Overall, two items (item 16 and item 20 of scale B) were unsatisfactory on both infit/outfit and DIF statistics; in each scale there were 6 or 7 well performing items. A content analysis of unfitting items was performed, but since problematic items were few and they were crucial to the instrument, all were retained.

### Criterion validity

The Strange Stories (administered to 115 subjects, i.e., 74% of the total sample) scores were used as an independent ToM measure to assess the criterion validity of Th.o.m.a.s. In terms of percentage of correct answers to all the six tasks, the adults performed better than the adolescents. The difference between the two percentages (68.6% for the adults, 48.8% for the adolescents) was statistically significant [*t*-test for unequal variances, *t*_(63)_ = −2.03, *p* = 0.046].

As shown in Table 4, only Scale B (Other–Self), i.e., the scale investigating third-person ToM in an egocentric perspective, correlated positively with the Strange Stories. This correlation was statistically significant both when the unpartialized coefficient was used and when the correlation was adjusted for age and education.

**Table 4:** **Pearson correlations between Th.o.m.a.s. scales and the Strange Stories scores**.

	**Scale A I–Me First-order first-person ToM**	**Scale B Other–Self, Allocentric third-person ToM**	**Scale C I–Other, Egocentric third-person ToM**	**Scale D Other–Me, Second-order (first-person) ToM**
Unpartialized	0.136	0.229^*^	0.126	0.119
Partialized for age and education	0.071	0.191^*^	0.056	0.056

* *p < 0.05*.

As regards the difference between the means of preadolescents/adolescents and those of the adults, the MANOVA analysis yielded statistical significance for both the omnibus F statistics and the four univariate *F* test (Table 5)^4^.

**Table 5:** **MANOVA results on preadolescents/adolescents vs. adults difference in means on the four Th.o.m.a.s. scales**.

**Scale**	**Preadolescents and adolescents^a^ (*N* = 80)**	**Adults^a^ (*N* = 74)**	**Univariate F statistics and η^2^**
A I–Me, First-order first-person ToM	3.21 (0.48)	3.82 (0.25)	*F*_(1, 152)_ = 93.73; *p* < 0.0001, 0.38
B Other–Self, Allocentric third-person ToM	2.97 (0.46)	3.70 (0.36)	*F*_(1, 152)_ = 116.41; *p* < 0.0001, 0.43
C I–Other, Egocentric third-person ToM	2.94 (0.51)	3.71 (0.35)	*F*_(1, 152)_ = 117.98; *p* < 0.0001, 0.43
D Other–Me, Second-order first-person ToM	2.98 (0.49)	3.66 (0.35)	*F*_(1, 152)_ = 95.70; *p* < 0.0001, 0.38
			

a *Mean and (standard deviation); Multivariate F statistics associated to Pillai's trace: F_(4, 149)_ = 35.57; p < 0.001*.

## Discussion

The Th.o.m.a.s. (Bosco et al., 2009; see Appendix A in Supplementary Material) is a semi-structured interview investigating Theory of Mind (ToM). The 37 open-ended questions of which it is comprised are organized in four scales, called A (I–Me), B (Other–Self), C (I–Other), and D (Other–Me), each focusing on one of the knowledge domains in which ToM manifests itself. The questions leave the interviewee free to articulate her thoughts; she is also invited to propose examples taken from her own biography or anyway from the real world, and thus to make her understanding of the mental states both of her own and of the others explicit and to reflect upon them. Th.o.m.a.s has been administered to persons with a diagnosis of schizophrenia (Bosco et al., 2009), sex offenders (Castellino et al., 2011), persons suffering from alcohol abuse (Bosco et al., 2014a), persons with congenital heart disease (Chiavarino et al., 2015), and persons with bulimia (Laghi et al., 2014). In each of these cases Th.o.m.a.s. has proved a useful clinical tool able to discriminate between healthy control and non-healthy participants.

The aim of this study was to assess the validity and the reliability of the Th.o.m.a.s. scores. In particular inter-rater agreement, internal consistency, dimensional structure, items' functioning, and criterion validity were evaluated in a sample of 156 healthy adolescents, and adults.

Internal consistency of the scores in the four scales composing Th.o.m.a.s. ranged from good to really good as defined in the literature (De Vellis et al., 1991). Reliability was satisfactory also when evaluated by Partial credit model. The inter-rater agreement was acceptable, ranging from fair to moderate (Shrout, 1998).

The dimensional structure of the Th.o.m.a.s. scores was explored with both CFA and Rasch analysis, yielding divergent results. The CFA model representing the four theoretical scales fitted the data very well, but factors were highly correlated and a more parsimonious two factors model fitted the data equally well. Correlation was also very high (0.96) in the latter model, which might suggest that a single broader ToM dimension existed. By contrast, the PCA of model residuals in the Partial credit model analysis showed that the 37 items of the instrument were not indicators of a single latent construct, but belonged to four distinct scales, corresponding to those that were theoretically expected.

As reported in literature, factor analysis and Rasch modeling can produce divergent results in terms of dimensionality under specific conditions regarding, for example, the proportion of items per dimension, the level of correlation between dimensions, and a non-linear relationship between items scores and the latent dimension (McDonald, 1965; Smith, 1996; Waugh and Chapman, 2005; Yu et al., 2007). The reason for the discrepancy between CFA and Partial credit model in our study lies most likely in the high correlation between the four scale scores. As shown in a simulation study by Smith (1996), Rasch analysis works better than factor analysis when dimensions are highly correlated and worse when correlations are low. Moreover, Rasch analysis, which does not rely upon correlations, is preferable to factor analysis when the variables are not continuous (Boone et al., 2014). In the light of these remarks, and according to Rasch results, Th.o.m.a.s. can be considered an instrument assessing four distinct, even if highly correlated, dimensions of ToM.

The high level of correlation between the dimensions scores deserves further consideration. A certain amount of correlation is theoretically expected, since the four dimensions are components of a broader construct, namely ToM abilities; however, the level of correlation was probably inflated due to some methodological features: (i) the uniformity of the test structure, which is entirely composed of open-ended questions; (ii) the persistence of the same persons as raters; and (iii) the uniformity of the contents investigated (all the scales assess mental states related to beliefs, emotions and desires). Furthermore, in healthy adults these different dimensions of ToM are substantially well integrated (which may not be the case in clinical populations), producing high scores overall the four scales. Younger people obtained lower scores, which might also have contributed to the inflation of correlations (Bewick et al., 2003).

Overall, the performance of the Partial credit models in each of the four scales was satisfactory. Only six items out of 37 showed poor fitting and scales resulted to be partially invariant with respect to age and gender. In fact, only in few cases item locations (difficulties) were not the same between adolescents and adults with the same person location (ability) or between male and female with the same person location.

Regarding the comparison between mean scales scores, the only significant difference found was that the sample performed better to Scale A (I–Me) with respect to B (Other–Self) and C (I–Other). This is in line with other studies in the literature to the effect that first-person ToM is generally the easiest to handle (see also Lysaker et al., 2005). The sample also performed better to Scale A (I–Me) than D (Other–Me). This is again in line with the literature, according to which healthy children find first-order ToM tasks easier than second-order ones (Perner and Wimmer, 1985; the two types of tasks are respectively explored in Scales A and D of Th.o.m.a.s.). This is also the case in clinical samples: first-order ToM is easier than second-order to persons with a diagnosis of schizophrenia, both when evaluated with Th.o.m.a.s. (Bosco et al., 2009) and with other classic false-belief tasks (e.g., Mazza et al., 2001). Instead, the difference between allocentric and egocentric third-person ToM has remained quite unexplored in the literature about mentalizing. A previous study using Th.o.m.a.s. in sex offenders (Castellino et al., 2011) found that they performed worse on Scale B (allocentric) than C (egocentric third-person ToM), showing that the comparison between the two perspectives may be interesting in some cases. However, further studies with clinical samples are necessary in order to investigate this issue.

We employed the Strange Stories task as an independent ToM measure to analyze criterion validity. Statistical analysis showed that it correlated positively with Scale B (Other–Self: allocentric third-person ToM), but not with the other three Th.o.m.a.s. scales. This is as expected, since the Strange Stories task measures third-person, allocentric ToM.

Finally, MANOVA results confirmed the expectation that the scores would increase from adolescents to adults, thus adding further evidence to the idea that the development of ToM continues during childhood, through adolescence (Choudhury et al., 2006; Bosco et al., 2014b; Brizio et al., 2015) and into adulthood (Maylor et al., 2002; Dumontheil et al., 2010).

In conclusion, our results supported the theoretical distinction among the four scales. Despite the strong correlations between them in the present sample of healthy people, they should not be considered secondary dimensions of a broader but homogeneous ToM factor or treated as source of noise in the data. Actually, at least two theoretically sound features emerged, namely that Scale A (I–Me) is easier than the others and that only Scale B (Other–Self) was correlated to a third-person ToM test, the Strange Stories task. This conclusion is also supported by previous researches finding different patterns of performance on the four scales in different clinical samples (Laghi et al., 2014; Chiavarino et al., 2015).

Future research directions basically coincide with the attempts to overcome the current limitations of Th.o.m.a.s. and its use. First, the size of the healthy sample ought to be steadily increased from the current figure of 156. Furthermore, it will be necessary to provide the normative data for the Italian population and to administer additional ToM tests beyond the Strange Stories to provide further empirical evidence on construct validity (see for example Brüne et al., 2016).

It will also be necessary to understand the cultural properties of Th.o.m.a.s., that is the extent to which it is embedded in how native Italians, or Europeans, or Westerners conceive of ToM, or in universal features of human social cognition. Of course, this might then yield modifications either in the instrument itself or at least in how scores would be given to members of different cultures.

Still another direction of development which can be expected to yield interesting results is the use of Th.o.m.a.s. with different types of clinical populations. Those in which it has already been employed (namely, to repeat, schizophrenia, criminal sexual behaviors, alcohol abuse, congenital heart disease, and bulimia) do exhibit differences in their respective profiles of ToM (mal)functioning. Given the importance of ToM in our species, its delicacy, and its dependence on individual and contextual factors, this comes as no surprise; it is analogously reasonable to expect further differences to be found in other conditions of clinical interest.

### Conflict of interest statement

The authors declare that the research was conducted in the absence of any commercial or financial relationships that could be construed as a potential conflict of interest.

## Footnotes

^1^Previous versions of the tool included 39 questions; in the final version, two were dropped because they turned out to be redundant.

^2^In particular, the ICC type C1, which measures the absolute agreement in a two-way random analysis of variance model for average measures, was adopted (McGraw and Wong, 1996).

^3^As an additional check on the validity of the results of the parametric analysis a non parametric MANOVA (Finch, 2005) and the Mann-Whitney test were performed.

^4^The nonparametric MANOVA and the Mann-Whitney test statistics also resulted in statistically significant differences.
