# Supplementary material for: Psychometric Properties of the Theory of Mind Assessment Scale in a Sample of Adolescents and Adults
Source: Front Psychol. 2016 May 9;7:566. doi: 10.3389/fpsyg.2016.00566 (PMC4860419; doi:10.3389/fpsyg.2016.00566)
Supplement: Supplementary file 1 [file DataSheet1.docx]

**APPENDIX A**

**Theory of Mind Assessment Scale (Th.o.m.a.s.)**

In the present Appendix, items are listed according to the division of Th.o.m.a.s. into subscales, in order to allow the reader to follow the theoretically-based order in which they are discussed in the article. The actual order of presentation during the interview is: 1-1a-2- 31-31a-32-11-11a-12-21-21a-22-3-3a-4-5-6-6a-33-33a-35-35a-34-37-38-13-13a-14-14a-15-15a-16-16a-23-23a-24-26-27-28-36-7-7a-8-8a-9-10-39-29-17-17a-18-19-20.

*A.1. Scale A (I–Me)*

[1] Do you happen to experience emotions that make you feel good? What? On what occasion?

Can you give an example?

[1a] (If the answer is negative) Why not?

[2] When you feel good, does that make any difference to you? What are the differences? Can you give an example of how you act or think, or of things that happen to you when you feel good?

[3] Do you happen to experience emotions that make you feel bad? What? On what occasions? Can you give an example?

[3a] (If the answer is negative) Have you ever asked yourself why?

[4] When you feel bad, does that make any difference to you? What are the differences? Can you give an example of how you act or think, or of things that happen to you when you feel bad?

[5] When you feel bad, do you feel you understand why? Can you give an example?

[6] Can you change your mood, when you want to? How? On what occasions? Can you give me an example?

[6a] (If the answer is negative) Why not?

[7] Do you happen to have wishes, and know what you want? What? On what occasions? Can you give an example?

[7a] (If the answer is negative) Do you ever ask yourself why?

[8] Do you try to fulfil your wishes? How? On what occasions? Can you give an example?

[8a] (If the answer is negative) Why not?

[9] Do you succeed in getting what you want? How? On what occasions? Can you give an example?

[10] Can you explain why you succeed/do not succeed?

*A.2. Scale B (Other–Self)*

[11] Do the other persons happen to experience emotions that make them feel good? What? On what occasions? Can you give an example?

[11a] (If the answer is negative) Why not, in your opinion?

[12] When the others feel good, does that make any difference to them? What differences does it make? Can you give an example of how they act or think, or of things happening to them when they feel good?

[13] And do the other persons happen to experience emotions that make them feel bad? What? On what occasions? Can you give an example?

[13a] (If the answer is negative) Why not, in your opinion?

[14] When the others feel bad, does that make any difference to them? What differences does it make? Can you give an example of how they act or think, or of things happening to them when they feel bad?

[15] In your opinion, when the others feel bad, do they understand why? Can you give an example?

[15a] (If the answer is negative) Why don't they understand, in your opinion?

[16] And, in your opinion, can the others change their mood when they want to? How? On what occasions? Can you give an example?

[16a] (If the answer is negative) Why not, in your opinion?

[17] Do the others happen to have desires and know what they want? What sorts of desires do they have? Can you give an example?

[17a] (If the answer is negative) Why not, in your opinion?

[18] Do the others try to fulfil their desires? How? On what occasions? Can you give an example?

[18a] (If the answer is negative) Why don't they try, in your opinion?

[19] In your opinion, do the others succeed in getting what they want? How? On what occasions? Can you give an example?

[20] Why do/don't they succeed, in your opinion?

*A .3. Scale C (I–Other)*

[21] Do you notice when the others feel good? When does that happen? Can you give an example?

[21a] (If the answer is negative) Why don't you notice?

[22] When you notice that another person feels good, does that make any difference to you? What differences does it make? Can you give an example, of how you act or think, or of the things that happen to you?

[23] Do you notice when the others feel bad? When do you notice that? Can you give an example?

[23a] (If the answer is negative) Why don't you notice?

[24] When you notice that another person feels bad, does that make any difference to you? What differences does it make? Can you give an example of how you act or think, or of the things that happen to you?

[25] When the others feel bad, do you understand why? Can you give an example?

[25a] (If the answer is negative) Why can't you explain why other people feel bad?

[26] Do you ever want to influence the mood of the others? How? On what occasions? Can you give an example?

[27] Do you succeed in doing so? How? On what occasions? Can you give an example?

[28] How do you explain the fact that you manage/do not manage to do so?

[29] Do you think you understand the others’ wishes? What sort of wishes do they have? Can you give an example?

*A.4. Scale D (Other–Me)*

[31] Do the others notice when you feel good? When do they notice? Can you give an example?

[31a] (If the answer is negative) Why don't they notice?

[32] When the others notice that you feel good, does that make any difference to them? What difference does it make? Can you give an example of how they act or think when they notice that you feel good?

[33] Do the others notice when you feel bad? When do they notice? Can you give an example?

[33a] (If the answer is negative) Why don't they notice?

[34] When the others notice that you feel bad, does that make any difference to them? What difference does it make? Can you give an example of how they act or think when they notice that you feel bad?

[35] When you feel bad, do the others understand why? Can you give an example?

[35a] (If the answer is negative) Why don't they understand?

[37] Can the others influence your mood? How? On what occasions? Can you give an example?

[38] How do you explain that they succeed/do not succeed in doing so?

[39] Do you think that the others understand your desires? In your opinion, what sort of wishes do they think you have? Can you give an example?

**APPENDIX B**

**Rating criteria of the Theory of Mind Assessment Scale (Th.o.m.a.s.)**

The interview is recorded (with the interviewee’s permission) and transcribed; the replies are rated on the transcript. Each judge assigns each reply a score ranging from 0 to 4 and inserts it in the relevant cell; each cell thus corresponds to a reply given by the interviewee and represents the specific intersection between two of the dimensions investigated. See also Bosco et al., (2009) and Bosco et al., (2014) for examples of answers.

A score of 0 is attributed:

– when the interviewee remains silent, however encouraged by the interviewer;

– when the reply is incomprehensibly confused, or completely irrelevant to the question, or detached from reality.

A score of 1 is attributed:

– when the interviewee gains time without in fact providing any meaningful reply;

– when the interviewee says that she does not know how to reply or limits herself to replying yes or no without adding;

anything else, however encouraged by the interviewer;

– when the reply is confused or inconsistent with regard to the question;

– when an example is provided (spontaneously or after a request by the interviewer) which does not appear consistent with the reply itself.

A score of 2 is assigned to a reply which:

– is confused, albeit relevant to the question;

– is a mere repetition of the question without any further consideration or explanation (e.g., a tautological reply);

– expresses an emotional tone which is inconsistent with the question (e.g., an emotionally positive reply to a question concerning negative emotions);

– is not correctly aligned with the perspective required by the question, e.g., when the question concerns another person’s emotional states (allocentric perspective) and the reply only refers to the interviewee himself or herself (egocentric perspective).

A score of 3 is assigned to a reply which:

– is not articulated;

– is articulated and coherent, but provided with difficulty or only after several attempts on the part of the interviewer;

– is consistent with the question but has no concrete, meaningful example;

– provides an example which is approximate, generic, meaningless, or only refers to behaviors instead of mental states or events;

– is coherent and consistent, but generic, stereotyped or only slightly contextualized.

A score of 4 is attributed to a reply which:

– is coherent, detailed and organized, with significant, coherent and contextualized examples;

– refers in different ways to the interviewee’s own mental states and events and to those of the others, thus providing not a generic or prototypical reply, but a contextualized one which bears a relation to the interviewee’s personal experience.

To obtain a score of 4, it is not necessary for the interviewee to provide an example based on her personal experience: it is sufficient that the reply is contextualized in a well detailed manner, that there are differentiations; thus, an invented example may suffice if it is meaningful and well contextualized.

| **APPENDIX C – Pearson’s correlations between Th.o.m.a.s. items** | | | | | | | | | | | | | | | | | | | | | | | | | | | | | | | | | | | | | |
| --- | --- | --- | --- | --- | --- | --- | --- | --- | --- | --- | --- | --- | --- | --- | --- | --- | --- | --- | --- | --- | --- | --- | --- | --- | --- | --- | --- | --- | --- | --- | --- | --- | --- | --- | --- | --- | --- |
| Item N. | 5 | 10 | 7 | 8 | 9 | 1 | 2 | 6 | 3 | 4 | 15 | 20 | 17 | 18 | 19 | 11 | 12 | 16 | 13 | 14 | 25 | 28 | 29 | 26 | 21 | 22 | 27 | 23 | 24 | 35 | 38 | 39 | 31 | 32 | 37 | 33 | 34 |
| 5 |  |  |  |  |  |  |  |  |  |  |  |  |  |  |  |  |  |  |  |  |  |  |  |  |  |  |  |  |  |  |  |  |  |  |  |  |  |
| 10 | .47 |  |  |  |  |  |  |  |  |  |  |  |  |  |  |  |  |  |  |  |  |  |  |  |  |  |  |  |  |  |  |  |  |  |  |  |  |
| 7 | .59 | .53 |  |  |  |  |  |  |  |  |  |  |  |  |  |  |  |  |  |  |  |  |  |  |  |  |  |  |  |  |  |  |  |  |  |  |  |
| 8 | .58 | .51 | .54 |  |  |  |  |  |  |  |  |  |  |  |  |  |  |  |  |  |  |  |  |  |  |  |  |  |  |  |  |  |  |  |  |  |  |
| 9 | .58 | .61 | .46 | .60 |  |  |  |  |  |  |  |  |  |  |  |  |  |  |  |  |  |  |  |  |  |  |  |  |  |  |  |  |  |  |  |  |  |
| 1 | .56 | .50 | .52 | .48 | .57 |  |  |  |  |  |  |  |  |  |  |  |  |  |  |  |  |  |  |  |  |  |  |  |  |  |  |  |  |  |  |  |  |
| 2 | .47 | .36 | .39 | .32 | .35 | .52 |  |  |  |  |  |  |  |  |  |  |  |  |  |  |  |  |  |  |  |  |  |  |  |  |  |  |  |  |  |  |  |
| 6 | .40 | .39 | .50 | .36 | .24 | .43 | .40 |  |  |  |  |  |  |  |  |  |  |  |  |  |  |  |  |  |  |  |  |  |  |  |  |  |  |  |  |  |  |
| 3 | .56 | .48 | .43 | .40 | .42 | .55 | .44 | .45 |  |  |  |  |  |  |  |  |  |  |  |  |  |  |  |  |  |  |  |  |  |  |  |  |  |  |  |  |  |
| 4 | .58 | .40 | .49 | .40 | .38 | .56 | .40 | .38 | .63 |  |  |  |  |  |  |  |  |  |  |  |  |  |  |  |  |  |  |  |  |  |  |  |  |  |  |  |  |
| 15 | .47 | .54 | .47 | .50 | .45 | .55 | .44 | .45 | .55 | .51 |  |  |  |  |  |  |  |  |  |  |  |  |  |  |  |  |  |  |  |  |  |  |  |  |  |  |  |
| 20 | .26 | .39 | .37 | .38 | .32 | .35 | .24 | .29 | .27 | .18 | .43 |  |  |  |  |  |  |  |  |  |  |  |  |  |  |  |  |  |  |  |  |  |  |  |  |  |  |
| 17 | .60 | .57 | .46 | .53 | .60 | .53 | .48 | .40 | .44 | .41 | .50 | .36 |  |  |  |  |  |  |  |  |  |  |  |  |  |  |  |  |  |  |  |  |  |  |  |  |  |
| 18 | .47 | .52 | .52 | .47 | .50 | .44 | .40 | .22 | .48 | .39 | .52 | .47 | .60 |  |  |  |  |  |  |  |  |  |  |  |  |  |  |  |  |  |  |  |  |  |  |  |  |
| 19 | .50 | .60 | .49 | .49 | .59 | .42 | .37 | .33 | .46 | .33 | .54 | .54 | .60 | .61 |  |  |  |  |  |  |  |  |  |  |  |  |  |  |  |  |  |  |  |  |  |  |  |
| 11 | .34 | .39 | .31 | .35 | .36 | .42 | .30 | .31 | .38 | .33 | .49 | .22 | .41 | .43 | .35 |  |  |  |  |  |  |  |  |  |  |  |  |  |  |  |  |  |  |  |  |  |  |
| 12 | .54 | .49 | .40 | .43 | .46 | .61 | .47 | .32 | .49 | .58 | .53 | .22 | .54 | .50 | .41 | .56 |  |  |  |  |  |  |  |  |  |  |  |  |  |  |  |  |  |  |  |  |  |
| 16 | .33 | .29 | .22 | .28 | .29 | .29 | .35 | .37 | .39 | .34 | .32 | .16 | .36 | .28 | .29 | .31 | .29 |  |  |  |  |  |  |  |  |  |  |  |  |  |  |  |  |  |  |  |  |
| 13 | .47 | .43 | .34 | .42 | .44 | .49 | .29 | .25 | .50 | .48 | .49 | .16 | .45 | .48 | .40 | .53 | .54 | .33 |  |  |  |  |  |  |  |  |  |  |  |  |  |  |  |  |  |  |  |
| 14 | .47 | .55 | .44 | .54 | .51 | .52 | .37 | .39 | .50 | .45 | .53 | .32 | .55 | .50 | .41 | .52 | .48 | .31 | .48 |  |  |  |  |  |  |  |  |  |  |  |  |  |  |  |  |  |  |
| 25 | .55 | .56 | .54 | .52 | .56 | .60 | .53 | .43 | .55 | .47 | .57 | .35 | .56 | .55 | .51 | .45 | .53 | .44 | .43 | .51 |  |  |  |  |  |  |  |  |  |  |  |  |  |  |  |  |  |
| 28 | .45 | .50 | .52 | .47 | .41 | .52 | .42 | .50 | .43 | .41 | .51 | .37 | .47 | .39 | .31 | .35 | .36 | .26 | .28 | .46 | .54 |  |  |  |  |  |  |  |  |  |  |  |  |  |  |  |  |
| 29 | .50 | .54 | .44 | .49 | .54 | .56 | .46 | .38 | .59 | .55 | .61 | .32 | .68 | .60 | .59 | .42 | .50 | .26 | .52 | .55 | .59 | .42 |  |  |  |  |  |  |  |  |  |  |  |  |  |  |  |
| 26 | .50 | .44 | .43 | .40 | .37 | .50 | .43 | .42 | .47 | .45 | .50 | .37 | .47 | .40 | .39 | .41 | .48 | .33 | .47 | .42 | .47 | .54 | .49 |  |  |  |  |  |  |  |  |  |  |  |  |  |  |
| 21 | .47 | .29 | .28 | .33 | .37 | .50 | .46 | .40 | .44 | .44 | .46 | .30 | .48 | .36 | .36 | .45 | .46 | .37 | .39 | .44 | .51 | .35 | .46 | .52 |  |  |  |  |  |  |  |  |  |  |  |  |  |
| 22 | .45 | .33 | .40 | .37 | .36 | .46 | .51 | .37 | .49 | .46 | .53 | .36 | .41 | .51 | .41 | .46 | .53 | .32 | .40 | .49 | .50 | .38 | .51 | .42 | .48 |  |  |  |  |  |  |  |  |  |  |  |  |
| 27 | .38 | .43 | .45 | .35 | .37 | .47 | .39 | .34 | .29 | .29 | .40 | .24 | .42 | .39 | .38 | .37 | .38 | .26 | .29 | .36 | .48 | .42 | .45 | .55 | .38 | .47 |  |  |  |  |  |  |  |  |  |  |  |
| 23 | .45 | .48 | .40 | .43 | .51 | .51 | .34 | .27 | .53 | .44 | .55 | .42 | .53 | .56 | .52 | .38 | .49 | .27 | .51 | .53 | .54 | .45 | .57 | .38 | .41 | .49 | .33 |  |  |  |  |  |  |  |  |  |  |
| 24 | .58 | .51 | .55 | .51 | .55 | .62 | .43 | .38 | .46 | .50 | .54 | .24 | .55 | .41 | .41 | .35 | .50 | .41 | .46 | .46 | .61 | .49 | .49 | .52 | .41 | .48 | .49 | .48 |  |  |  |  |  |  |  |  |  |
| 35 | .58 | .56 | .56 | .61 | .61 | .60 | .55 | .39 | .61 | .61 | .58 | .42 | .55 | .61 | .58 | .46 | .59 | .41 | .51 | .59 | .67 | .49 | .58 | .48 | .42 | .52 | .43 | .58 | .52 |  |  |  |  |  |  |  |  |
| 38 | .43 | .38 | .43 | .39 | .39 | .49 | .42 | .37 | .44 | .51 | .37 | .20 | .29 | .27 | .22 | .25 | .43 | .29 | .30 | .40 | .43 | .45 | .28 | .33 | .28 | .38 | .25 | .33 | .45 | .49 |  |  |  |  |  |  |  |
| 39 | .47 | .50 | .40 | .44 | .49 | .46 | .41 | .30 | .40 | .35 | .48 | .32 | .64 | .55 | .53 | .34 | .43 | .31 | .36 | .47 | .47 | .46 | .59 | .42 | .37 | .41 | .43 | .48 | .48 | .49 | .25 |  |  |  |  |  |  |
| 31 | .35 | .49 | .32 | .31 | .36 | .41 | .41 | .32 | .30 | .32 | .51 | .24 | .44 | .33 | .42 | .44 | .41 | .27 | .40 | .47 | .39 | .32 | .45 | .36 | .33 | .37 | .47 | .34 | .38 | .35 | .23 | .44 |  |  |  |  |  |
| 32 | .52 | .45 | .53 | .44 | .38 | .48 | .50 | .42 | .35 | .42 | .62 | .33 | .48 | .40 | .51 | .46 | .49 | .28 | .39 | .50 | .48 | .39 | .44 | .49 | .54 | .44 | .43 | .39 | .43 | .48 | .30 | .43 | .65 |  |  |  |  |
| 37 | .52 | .45 | .44 | .39 | .40 | .52 | .50 | .45 | .45 | .49 | .46 | .30 | .41 | .33 | .41 | .31 | .44 | .35 | .27 | .43 | .62 | .37 | .42 | .45 | .44 | .43 | .41 | .48 | .53 | .58 | .52 | .44 | .29 | .41 |  |  |  |
| 33 | .55 | .45 | .44 | .46 | .44 | .48 | .39 | .30 | .47 | .51 | .43 | .24 | .43 | .54 | .43 | .33 | .55 | .37 | .39 | .48 | .56 | .38 | .46 | .39 | .39 | .31 | .35 | .49 | .47 | .62 | .40 | .35 | .36 | .38 | .44 |  |  |
| 34 | .56 | .51 | .54 | .49 | .57 | .51 | .36 | .32 | .39 | .53 | .53 | .26 | .46 | .43 | .40 | .37 | .47 | .27 | .42 | .52 | .56 | .50 | .54 | .50 | .38 | .43 | .56 | .51 | .59 | .55 | .29 | .47 | .44 | .51 | .45 | .45 |  |
| M | 3.4 | 3.4 | 3.7 | 3.68 | 3.6 | 3.6 | 3.3 | 3.5 | 3.6 | 3.6 | 3.1 | 3.5 | 3.3 | 3.5 | 3.4 | 3.3 | 3.2 | 3.3 | 3.4 | 3.4 | 3.2 | 3.4 | 3.2 | 3.4 | 3.3 | 3.3 | 3.3 | 3.4 | 3.4 | 3.2 | 3.5 | 3.2 | 3.2 | 3.0 | 3.4 | 3.5 | 3.4 |
| SD | .88 | .69 | .55 | .66 | .67 | .59 | .83 | .71 | .64 | .59 | .96 | .68 | .94 | .73 | .76 | .75 | .78 | .79 | .75 | .74 | .93 | .77 | .93 | .69 | .80 | .81 | .76 | .72 | .76 | .86 | .62 | .92 | .84 | .91 | .70 | .61 | .72 |
